# Is SARS-CoV-2 originated from laboratory? A rebuttal to the claim of formation via laboratory recombination

**DOI:** 10.1080/22221751.2020.1738279

**Published:** 2020-03-08

**Authors:** Pei Hao, Wu Zhong, Shiyang Song, Shiyong Fan, Xuan Li

**Affiliations:** a Key Laboratory of Molecular Virology and Immunology, Institute Pasteur of Shanghai, Center for Biosafety Mega-Science, Chinese Academy of Sciences, Shanghai, People’s Republic of China; b National Engineering Research Center For the Emergence Drugs, Beijing Institute of Pharmacology and Toxicology, Beijing, People’s Republic of China; c Key Laboratory of Synthetic Biology, CAS Center for Excellence in Molecular Plant Sciences, Chinese Academy of Sciences, Shanghai, People’s Republic of China

Dr James Lyons-Weiler, the CEO of the “Institute for Pure and Applied Knowledge,” made an appalling online statement on 3 February 2020, which claimed the novel coronavirus (SARS-CoV-2) responsible for the ongoing COVID-19 epidemic was most likely constructed via laboratory recombination [1]. In the results, he showed SARS-CoV-2 had a unique inserted sequence (1378 bp) located in the middle of its spike glycoprotein gene that had no match in other coronaviruses (Supplementary Figure 1). Furthermore, he claimed this unique sequence was similar to some sequence in pShuttle-SN (Supplementary Figure 2), a common expression vector used in research laboratory.

To check on his claim, we ran a thorough analysis on his results, and found some serious mistakes in his distorted analysis. Thus, we drew an opposite conclusion that there was no evidence to support the theory for the formation of SARS-CoV-2 in a laboratory.

First of all, this unique sequence is not specific to SARS-CoV-2. By aligning several coronaviruses discovered from natural sources, our result showed that this “unique” sequence (1378 bp) from SARS-CoV-2 was also found in other coronavirus (Figure 1), with a high sequence identity. This indicated that this particular fragment in SARS-CoV-2 spike gene was widely spread in naturally existing coronaviruses and was not from laboratory.

**Figure 1.**
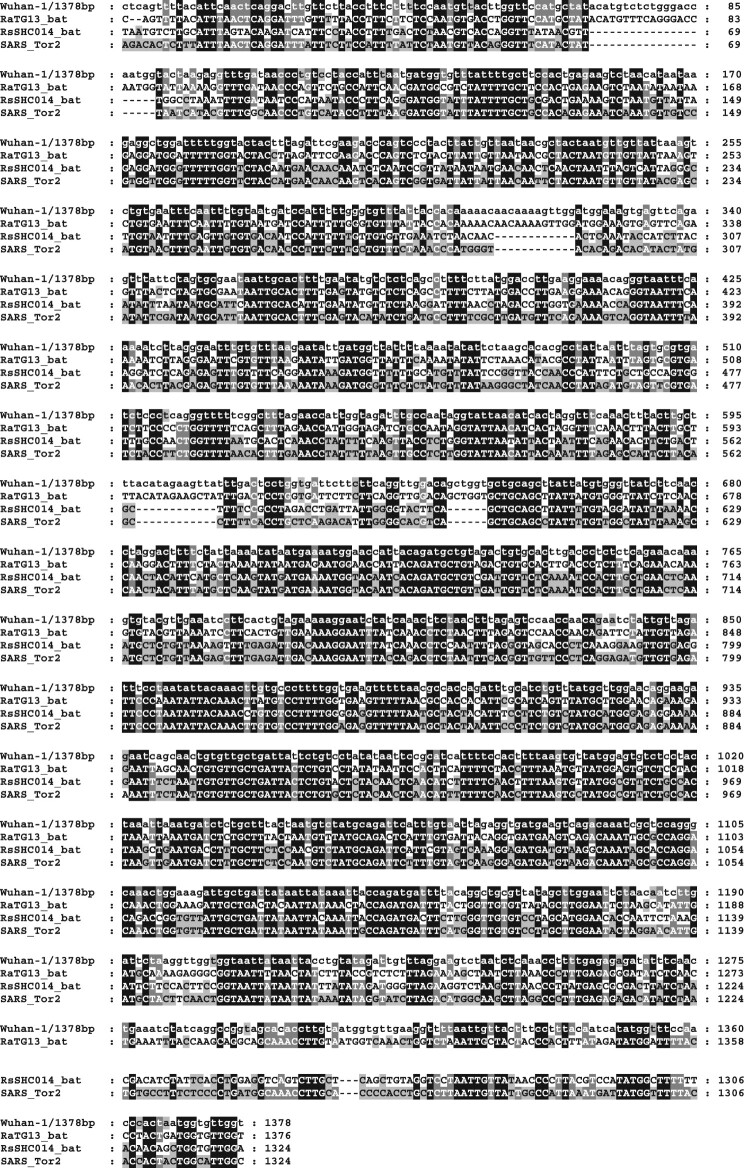
The 1378 bp fragment of SARS-CoV-2 spike gene (claimed by James Lyons-Weiler to be unique in SARS-CoV-2 and similar to some sequence in pShuttle-SN) is aligned with sequences from natural sources. Wuhan-1/1378bp, the SARS-CoV-2 spike gene fragment (1378 bp) claimed by James Lyons-Weiler; RaTG13_bat and RsSHC014_bat, bat coronavirus sequences; SARS_Tor2, SARS-CoV spike gene sequence. (Note: Alignment of the 1378 bp fragment of SARS-CoV-2 spike gene to the sequence in pShuttle-SN is shown in Supplementary Figure 2).

Second, how should we explain the sequence similarity between this SARS-CoV-2 spike gene fragment and the sequence in pShuttle-SN? This is indeed another misleading statement from James Lyons-Weiler, who described pShuttle-SN as a vector. We carefully analysed the source of pShuttle-SN, and realized that pShuttle-SN was built in 2005 as an expression plasmid carrying sequence of spike gene from SARS-CoV, the coronavirus responsible for 2003 SARS epidemic [2]. The pShuttle-SN should not be called as a vector but a plasmid generated from Adeno-X^TM^ to study SARS-CoV. The real empty vector was Adeno-X^TM^ expression system (Clontech Laboratories, Inc.), which had no significant homology to any part of the genome of SARS-CoV-2. Since pShuttle-SN had a fragment of the spike gene from SARS-CoV, which was similar to that from SARS-CoV-2, it was no wonder the SARS-CoV-2 spike gene fragment (1378 bp) was found to match with some sequence in pShuttle-SN. On an added note, our results indicated its sequence similarity to the pShuttle-SN fragment (Supplementary Figure 2) was lower than to the natural coronaviral sequences (Figure 1).

In conclusion, we found the so-called unique sequence (1387 bp) in the SARS-CoV-2 spike gene was widely available in coronavirus from natural source, opposite to what James Lyons-Weiler had claimed. The pShuttle-SN plasmid contained a fragment from the spike gene of SARS-nCoV, which caused the similarity match between it and the SARS-CoV-2 spike gene sequence. We call upon Dr James Lyons-Weiler to take his responsibility to make a public correction to his un-supported claim.

## Supplementary Material

Supplemental Material
