# Supplementary material for: Is SARS-CoV-2 originated from laboratory? A rebuttal to the claim of formation via laboratory recombination
Source: Emerg Microbes Infect. 2020 Mar 8;9(1):545–7. doi: 10.1080/22221751.2020.1738279 (PMC7144200; doi:10.1080/22221751.2020.1738279)
Supplement: Supplemental Material [file TEMI_A_1738279_SM5849.zip › Supplementary-figure-1_v3.doc]

Supplementary Figure 1.

Original data from James Lyons-Weiler’s web site [1]: the resulting massive alignment confirms a major unique inserted element in 2019-nCoV not found in other bat coronaviruses, nor in SARS in the homologous genomic position.

Reference:

1: <https://jameslyonsweiler.com/2020/02/02/moderately-strong-confirmation-of-a- laboratory-origin-of-2019-ncov/>
