# Supplementary material for: Is SARS-CoV-2 originated from laboratory? A rebuttal to the claim of formation via laboratory recombination
Source: Emerg Microbes Infect. 2020 Mar 8;9(1):545–7. doi: 10.1080/22221751.2020.1738279 (PMC7144200; doi:10.1080/22221751.2020.1738279)
Supplement: Supplemental Material [file TEMI_A_1738279_SM5849.zip › Supplementary-figure-2.doc]

```
1 ########################################
2 # Program: water
3 # Rundate: Wed  5 Feb 2020 02:32:15
4 # Commandline: water
5 # 	-auto
6 # 	-stdout
7 # 	-asequence emboss_water-I20200205-023213-0325-67015509-p1m.asequence
8 # 	-bsequence emboss_water-I20200205-023213-0325-67015509-p1m.bsequence
9 # 	-datafile EBLOSUM62
10 # 	-gapopen 10.0
11 # 	-gapextend 0.5
12 # 	-aformat3 pair
13 # 	-sprotein1
14 # 	-sprotein2
15 # Align_format: pair
16 # Report_file: stdout
17 ########################################
18
19 #=======================================
20 #
21 # Aligned_sequences: 2
22 # 1: 1
23 # 2: AY862402.1
24 # Matrix: EBLOSUM62
25 # Gap_penalty: 10.0
26 # Extend_penalty: 0.5
27 #
28 # Length: 1387
29 # Identity: 	916/1387 (66.0%)
30 # Similarity: 	916/1387 (66.0%)
31 # Gaps: 	71/1387 ( 5.1%)
32 # Score: 4577.5
33 #
34 #=======================================
35
36 1 	2 tcagt---tttacattcaactcaggacttgttcttacctttcttttccaa	48
37 	||||. 	|.|..|||.|||||||||.||.||..|.||.||.|.|||.||
38 AY862402.1 	1134 TCAGACACTCTTTATTTAACTCAGGATTTATTTCTTCCATTTTATTCTAA	1183
39
40 1 	49 tgttacttggttccatgctatacatgtctctgggaccaatggtactaaga	98
41 	||||||..||||.|||.||||..|| 	|| 	|||
42 AY862402.1 	1184 TGTTACAGGGTTTCATACTATTAAT-----------CA----TAC-----	1213
43
44 1 	99 ggtttgataaccctgtcctaccatttaatgatggtgtttattttgcttcc	148
45 	||||||.|||||||||.||||.|||||.||||||.|||||||||||.||
46 AY862402.1 	1214 -GTTTGACAACCCTGTCATACCTTTTAAGGATGGTATTTATTTTGCTGCC	1262
47
48 1 	149 actgagaagtctaacataataagaggctggatttttggtactactttaga	198
49 	||.|||||.||.||..|..|..|.||.|||.||||||||.||||..|..|
50 AY862402.1 	1263 ACAGAGAAATCAAATGTTGTCCGTGGTTGGGTTTTTGGTTCTACCATGAA	1312
51
52 1 	199 ttcgaagacccagtccctacttattgttaataacgctactaatgttgtta	248
53 	....|||.|.|||||..|..|||||.||||.||..|||||||||||||||
54 AY862402.1 	1313 CAACAAGTCACAGTCGGTGATTATTATTAACAATTCTACTAATGTTGTTA	1362
55
56 1 	249 ttaaagtctgtgaatttcaattttgtaatgatccatttttgggtgtttat	298
57 	|...||..|||.|.|||.||||.|||.|..|.||.||.||.|.|||||.|
58 AY862402.1 	1363 TACGAGCATGTAACTTTGAATTGTGTGACAACCCTTTCTTTGCTGTTTCT	1412
59
60 1 	299 taccacaaaaacaacaaaagttggatggaaagtgagttcagagtttattc	348
61 	.|.|.|| 	|||.|..|.||..|..|...|...|||||
62 AY862402.1 	1413 AAACCCA--------------TGGGTACACAGACACATACTATGATATTC	1448
63
64 1 	349 --tagtgcgaataattgcacttttgaatatgtctctca-gccttttctta	395
65 	||.|||...||||||||||||.||.||..|.|||.|  ||||||||...
66 AY862402.1 	1449 GATAATGCATTTAATTGCACTTTCGAGTACATATCTGATGCCTTTTCGCT	1498
67
68 1 	396 tggaccttgaaggaaaacagggtaatttcaaaaatcttagggaatttgtg	445
69 	| ||..||..||.|||....||||||||.|||.|..|..|.||.||||||
70 AY862402.1 	1499 T-GATGTTTCAGAAAAGTCAGGTAATTTTAAACACTTACGAGAGTTTGTG	1547
71
72 1 	446 tttaagaatattgatggttattttaaaatatattctaagcacac-gccta	494
73 	|||||.||||..|||||.|.|.|..|..|.|| |....||...| .||||
74 AY862402.1 	1548 TTTAAAAATAAAGATGGGTTTCTCTATGTTTA-TAAGGGCTATCAACCTA	1596
75
76 1 	495 ttaatttagtgcgtgatctccctcagggtttttcggctttagaaccattg	544
77 	|..||.||||.||||||||.|||...||||||....||||..||||..|.
78 AY862402.1 	1597 TAGATGTAGTTCGTGATCTACCTTCTGGTTTTAACACTTTGAAACCTATT	1646
79
80 1 	545 gtagatttgccaataggtattaacatcactaggtttcaaactttacttgc	594
81 	.|..|.|||||..|.|||||||||||.||.|..|||..|.|..|.|||.|
82 AY862402.1 	1647 TTTAAGTTGCCTCTTGGTATTAACATTACAAATTTTAGAGCCATTCTTAC	1696

83
84
85
86
87
88
89
90
91
92
93
94
95
96
97
98
99
100
101
102
103
104
105
106
107
108
109
110
111
112
113
114
115
116
117
118
119
120
121
122
123
124
125
126
127
128
129
130
131
132
133
134
135
136
137
138
139
140
141
142
143
144
145
146
147
148
149
150
151
152
153
154


155
156
157
158
```

```
1 	595 tttacatagaagttatttgactcctg-gtgattcttcttcaggttggaca	643
||...|||  |.|||| |..|..| .|||..||...|.||
AY862402.1 	1697 ----------AGCCTTTT--CACCTGCGCAAGAC-ACTTGGGGCACGTCA	1733

1 	644 gctggtgctgcagcttattatgtgggttatcttcaacctaggacttttct	693
||||||||.||||.|||.||.|||.|..|.||.|..||.|||.|
AY862402.1 	1734 ------GCTGCAGCCTATTTTGTTGGCTATTTAAAGCCAACTACATTTAT	1777

1 	694 attaaaatataatgaaaatggaaccattacagatgctgtagactgtgcac	743
..|.||.|||.||||||||||.||.||.|||||||||||.||.|||.|.|
AY862402.1 	1778 GCTCAAGTATGATGAAAATGGTACAATCACAGATGCTGTTGATTGTTCTC	1827

1 	744 ttgaccctctctcagaaacaaagtgtacgttgaaatccttcactgtagaa	793
...|.||.||..|.|||...||.||..|..|.||...|||.....|.||.
AY862402.1 	1828 AAAATCCACTTGCTGAACTCAAATGCTCTGTTAAGAGCTTTGAGATTGAC	1877

1 	794 aaaggaatctatcaaacttctaactttagagtccaaccaacagaatctat	843
||||||||.||.||.||.|||||.||.||.||....||..|||.|..|.|
AY862402.1 	1878 AAAGGAATTTACCAGACCTCTAATTTCAGGGTTGTTCCCTCAGGAGATGT	1927

1 	844 tgttagatttcctaatattacaaacttgtgcccttttggtgaagttttta	893
|||.|||||.||||||||||||||||||||.||||||||.||.|||||||
AY862402.1 	1928 TGTGAGATTCCCTAATATTACAAACTTGTGTCCTTTTGGAGAGGTTTTTA	1977

1 	894 acgccaccagatttgcatctgtttatgcttggaacaggaagagaatcagc	943
|.||.||.|.|||..|.|||||.|||||.|||.|..|.||.|.|||....
AY862402.1 	1978 ATGCTACTAAATTCCCTTCTGTCTATGCATGGGAGGGAAAAAAAATTTCT	2027

1 	944 aactgtgttgctgattattctgtcctatataattccgcatcattttccac	993
||.||||||||||||||.|||||.||.||.||.||..|||..|||||.||
AY862402.1 	2028 AATTGTGTTGCTGATTACTCTGTGCTCTACAACTCAACATTTTTTTCAAC	2077

1 	994 ttttaagtgttatggagtgtctcctactaaattaaatgatctctgcttta	1043
.||||||||.|||||.||.|||.|.|||||.||.||||||||.|||||..
AY862402.1 	2078 CTTTAAGTGCTATGGCGTTTCTGCCACTAAGTTGAATGATCTTTGCTTCT	2127

1 	1044 ctaatgtctatgcagattcatttgtaattagaggtgatgaagtcagacaa	1093
|.|||||||||||||||||.||||||.|.|..||.|||||.||.||||||
AY862402.1 	2128 CCAATGTCTATGCAGATTCTTTTGTAGTCAAGGGAGATGATGTAAGACAA	2177

1 	1094 atcgctccagggcaaactggaaagattgctgattataattataaattacc	1143
||.||.|||||.||||||||....|||||||||||||||||||||||.||
AY862402.1 	2178 ATAGCGCCAGGACAAACTGGTGTTATTGCTGATTATAATTATAAATTGCC	2227

1 	1144 agatgattttacaggctgcgttatagcttggaattctaacaatcttgatt	1193
|||||||||.|..||.||.||..|.|||||||||.|||..||..|||||.
AY862402.1 	2228 AGATGATTTCATGGGTTGTGTCCTTGCTTGGAATACTAGGAACATTGATG	2277

1 	1194 ctaaggttggtggtaattataattacctgtatagattgtttagg-aagtc	1242
|||.......|||||||||||||||....|||||.|...||||. |.|.|
AY862402.1 	2278 CTACTCCAACTGGTAATTATAATTATAAATATAGGTATCTTAGACATGGC	2327

1 	1243 taatctcaaaccttttgagagagatatttcaactgaaatctatcaggcc-	1291
||.||.|..||.|||||||||||.||.||.|.||...  ||.||...||
AY862402.1 	2328 -AAGCTTAGGCCCTTTGAGAGAGACATATCTAATGTGC-CTTTCTCCCCT	2375

1 	1292 ggtagcacaccttgtaatggtgttgaaggttttaattgttactttccttt	1341
|.|.|||.||||||.|.......||. 	|.||||||||||.|..||.||
AY862402.1 	2376 GATGGCAAACCTTGCACCCCACCTGC---TCTTAATTGTTATTGGCCATT	2422

1 	1342 acaatcatatggtttccaacccactaatggtgttggt 	1378
|.|....||||||||..|..||||||.|||..|||||
AY862402.1 	2423 AAATGATTATGGTTTTTACACCACTACTGGCATTGGT 	2459

#---------------------------------------
#---------------------------------------


Supplementary Figure 2.
```

Original Data from James Lyons-Weiler's web site [1]: Blasting the novel sequence region against all non-viral sequences (to pick up vector technology) again results in pShuttle-SN. 1: the unique SARS-CoV-2 sequence (1378 bp) claimed by James Lyons-Weiler; AY862402.1: the sequence from pShuttle-SN.

Reference:

1:

https://jameslyonsweiler.com/2020/02/02/moderately-strong-confirmation-of-a-laboratory-origin-of-2019-ncov/
